# Predicting the Fate of Bisphenol A During Electrochemical Oxidation: A Simple Semiempirical Method Based on the Concentration Profile of Hydroxyl Radicals

**DOI:** 10.3390/ijms26104785

**Published:** 2025-05-16

**Authors:** Marija Ječmenica Dučić, Dragana Vasić Anićijević, Danka Aćimović, Ľubomír Švorc, Branko Bugarski, Radojica Pešić, Tanja Brdarić

**Affiliations:** 1 Department of Physical Chemistry, Vinča Institute of Nuclear Sciences—National Institute of the Republic of Serbia, University of Belgrade, Mike Petrovića Alasa 12–14, 11000 Belgrade, Serbia; 2 Institute of Analytical Chemistry, Faculty of Chemical and Food Technology, Slovak University of Technology in Bratislava, Radlinského 9, 812 37 Bratislava, Slovakia; 3 Department of Chemical Engineering, Faculty of Technology and Metallurgy, University of Belgrade, Karnegijeva 4, 11000 Belgrade, Serbia

**Keywords:** kinetic modelling, bisphenol A (BPA), electrooxidation processes, organic pollutants, second-order rate constants

## Abstract

The efficiency of electrochemical advanced oxidation processes (EAOPs) is fundamentally governed by hydroxyl-radical (•OH) generation. While direct experimental measurements of these transient species remain complex and impractical, robust computational methods for predicting their temporal profiles are notably scarce. This work presents a semi-empirical methodology based on H_2_O_2_ measuring experiments that enables indirect •OH quantification. We employed a recently developed carbon-based electrode and the priority pollutant bisphenol A (BPA) as the model system. The system achieved 92.3% BPA degradation with 84% mineralization efficiency during 5-h electrooxidation at 15 mA/cm^2^. Gas chromatography/mass spectrometry (GC/MS) was used for tracking BPA and detection of intermediates. On this basis, we developed a computational model that successfully predicts temporal concentration profiles of all reactive species interacting with •OH, along with degradation kinetics across current densities (10–20 mA/cm^2^). By incorporating predictions from the Toxicity Estimation Software Tool (T.E.S.T.), the developed model accurately simulates time-dependent evolution of relative toxicity throughout the treatment process. The presented approach has a general character and requires rather simple experimental input to predict and optimize degradation outcome in terms of input concentration, degradation time, current density, and final toxicity. Further modifications of the model would enable widening to other EAOPs systems.

## 1. Introduction

Electrochemical advanced oxidation processes (EAOPs) represent a promising and thoroughly investigated set of methods for the removal of organic pollutants in aqueous media [1,2]. During EAOPs, organic pollutants are depleted in interaction with active radicals generated on the electrode surface [3,4]. The type and concentration of radicals responsible for pollutant degradation depend on the electrode material and process conditions [5], and the efficiency of the process is, by the rule, directly proportional to the energy input [6,7,8]. The electrochemical oxidation is an EAOP method that uses electric current to oxidize organic matter, relying heavily on the generation and reactivity of hydroxyl radicals (•OH) [9,10,11]. Although other radicals such as hydroperoxyl radicals (HO_2_•) and sulfate radicals (SO_4_•^−^) also take place in sulfate-mediated electrochemical oxidation, the literature data report that their reactivity towards organic molecules is a few orders of magnitude lower compared to •OH [12,13,14,15]. Superoxide radicals (O_2_•^−^) also show lower reactivity compared to hydroxyl radicals (•OH) in wastewater treatment oxidation processes [16].

Due to their short lifespan and high reactivity, direct measurements of •OH radicals are challenging [17]. Several indirect methods have been developed to detect and quantify these elusive species, each with its own advantages and limitations. These include electron paramagnetic resonance (EPR) spectroscopy and fluorescence spectroscopy, which require complex equipment and are unreliable in oxidizing conditions [18,19]. Chemical dosimetry relies on the chemical reaction of •OH with specific probes to form measurable products, also being highly dependent on the probe selectivity and chemical properties of the system in general [19,20,21].

On the other hand, kinetic modelling provides insight into •OH radical concentration and enables understanding of its trends [22,23,24], once when the kinetics of •OH formation and depletion are known in reaction with other species, without external limitations of experimental measurements. They offer a complementary framework for understanding the complex processes governing •OH behavior [25], offering the possibility to further optimize experimental parameters while predicting transformation products and treatment efficiency [26]. Integrating experimental data with computational modeling enables the design of electrochemical systems for efficient and environmentally responsible pollutant degradation. Although it is generally accepted that active radicals should be considered when modelling degradation processes, studies involving general kinetic modelling of their concentration distribution during EAOPs remain scarce [27,28]. Existing research has primarily focused on photocatalytic UV/H_2_O_2_ systems [29,30], with particular attention to the degradation of pharmaceuticals [31,32,33], while highlighting the need to develop new models for compounds beyond dyes [34]. This gap is especially critical for harmful and ubiquitous priority pollutants that resist removal through conventional wastewater treatment methods and whose degradation bears the risk of the formation of potentially toxic by-products [35,36]. For these persistent contaminants, predicting both water toxicity and degradation product concentrations becomes particularly important [37]. In this view, semiempirical modelling of •OH radical behavior based on some simple experimental input is of particular interest, considering its universal role in EAOPs and AOPs in general.

For the reaction of the target organic pollutant with the •OH radical (Equation (1)), the general elementary second-order rate law can be written as the expression in Equation (2), where ${\left[Pollutant\right]}_{t}$ and ${\left[•OH\right]}_{t}$ are the time-dependent concentrations of pollutant and •OH radical, and $k_{•OH}^{pollutant}$ is a pseudo-second-order (i.e., binary or absolute) rate constant.

(1) $$Pollutant+•OH\;\overset{k_{r}}{\to }Products$$

(2) $$rate=-\frac{d{\left[Pollutant\right]}_{t}}{dt}=k_{•OH}^{pollutant}{\left[•OH\right]}_{t}{\left[Pollutant\right]}_{t}$$

In bulk solution, the effective concentration of •OH radicals available to degrade organic pollutants is affected by a number of coupled phenomena, such as electrode surface processes, adsorption, diffusion, and mixing [38,39]. Moreover, since •OH radicals are non-selective reactive species, they interact with the target organic pollutant, reaction intermediates and by-products, scavengers, and even with each other in recombination reactions, all causing ${\left[•OH\right]}_{t}$ to vary during the degradation process in a more or less complex manner [40].

In summary, the effective •OH radical concentration [41], acts as a specific characteristic for a particular electrode: a specific electrode generates a certain amount of •OH radicals, which, under the same transport conditions, leads to ${\left[•OH\right]}_{t}$ proportional to the applied current density *j* (mA/cm^2^).

Given the foregoing, there has been the need to develop a kinetic model that predicts the concentration profile of •OH radicals at different current densities, as a characteristic of a specific electrode, using simple experimental parameters as the input data. Once defined, the model would enable prediction of concentration distribution of pollutants and degradation intermediates depending on degradation time and current density. Moreover, known distribution of intermediates provides input for predicting related quantities of interest, such as degradation extent or total toxicity of the reaction mixture, when the toxicity parameters of the reaction intermediates are known.

This study employed bisphenol A (BPA), a well-characterized endocrine disruptor [42,43,44,45], as a model pollutant. While BPA degradation has been extensively studied in various advanced oxidation processes (e.g., photooxidation [46], catalytic wet air oxidation [36,47]), this work focuses on electrochemical oxidation using a SnO_2_-MWCNT@SS anode (tin oxide-modified multi-walled carbon nanotubes on stainless steel). All experimental data were generated using this specifically synthesized electrode, whose morphological and electrochemical properties were thoroughly characterized in our prior studies [48,49]. Mechanistic investigations [50,51,52] confirm that this composite anode mediates BPA degradation primarily via •OH radicals in sulfate media with a pH of 4–7 [53,54]. While BPA degradation—addressing process efficiency and operational parameters such as applied current density—were recently investigated in detail [49], a systematic analysis of intermediates formed during 5-h BPA electrooxidation has not been performed yet.

The aim of the present study was to bridge the required experimental gaps and integrate experimental data with kinetic modeling in order to predict concentration profiles and related quantities (degradation efficiency, degree of mineralization, and the toxicity of the sample during degradation) depending on given reaction parameters, such as current density and degradation time. To address the challenge of measuring •OH radicals necessary to obtain second-order rate constants, a strategy was to estimate ${\left[•OH\right]}_{t}$ using data on an easily measurable concentration of H_2_O_2_ which are in equilibrium with •OH radicals. A proposed approach was applied to the anodic oxidation of BPA using a SnO_2_-MWCNT@SS electrode under a current density of 15 mA/cm^2^. Experimental data on BPA degradation intermediates were collected using gas chromatography/mass spectrometry (GC/MS) analysis. The developed model was validated using data on BPA degradation at different current densities with the SnO_2_-MWCNT@SS electrode [49] and employed to predict time-dependent toxicity trends in the investigated degradation mixture. To evaluate its broader applicability to diverse organic pollutants, we further validated the model using rhodamine B degradation data obtained with the same SnO_2_-MWCNT@SS electrode system [48].

## 2. Results

### 2.1. Determination of Bisphenol A (BPA) Degradation Intermediates

The anode modeled in this study was comprehensively characterized in our prior work, including its synthesis procedure, structural/morphological properties, electrochemical performance, stability, and recyclability [48,49].

GC/MS analysis identified derivatized (silylated) intermediates during five-hour BPA electrolysis at 15 mA/cm^2^. Compound identification combined mass-to-charge ratios (m/z), characteristic ion fragmentation spectra from GC/MS analysis, and molecular formula matching using the NIST reference library [55]. This approach revealed six reactive intermediates, whose acute toxicities were quantified as 96-h LC_50_ values for *Pimephales promelas* (Fathead Minnow), as documented in Table 1.

**Figure 1:**
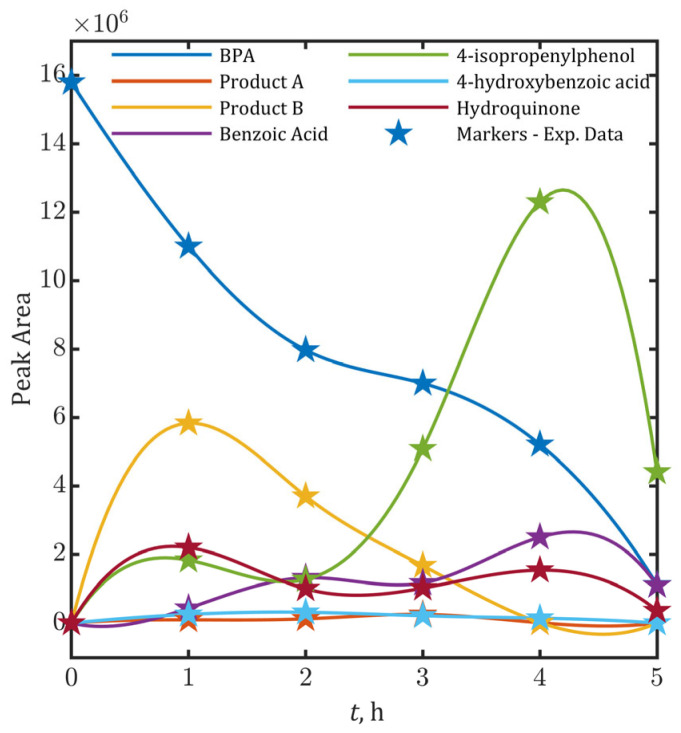
Temporal evolution of peak areas for bisphenol A (BPA) degradation intermediates during electrooxidation at 15 mA/cm^2^. Star markers (★) represent experimental data points.

All detected organic compounds have had aromatic structure, including either two aromatic rings or one ring, suggesting that oxidation of BPA begins at the 1-propane site, subsequently leading to the cleavage of ortho-positioned C-C bonds. While aliphatic intermediates were not detected—possibly due to the aromatic-targeted extraction method—their presence is inferred from the observed 84% mineralization efficiency (Table S2). Temporal evolution of relative intensities (peak area) for detected BPA degradation intermediates during the 5-h electrooxidation at 15 mA/cm^2^ is provided in Figure 1.

The results align with recent electro-Fenton (EF) studies of the same system under identical conditions [57]. While both processes reached high final efficiencies (EF: 96.3% BPA removal, 89% mineralization; electrooxidation, EO: 92.3% BPA removal, 84% mineralization), the kinetic advantage of EF was pronounced in the early stages of treatment. For example, at the 3-h mark, EF achieved 85.87% BPA degradation (vs. 68.24% for EO) and 79% mineralization (vs. 41.2% for EO).

### 2.2. Kinetic Model Framework

Based on previous results, we assumed the following steps in the degradation kinetics: (i) second-order •OH-driven BPA degradation (k_S_, Equation (3)) forms P_tr_ (lumped two-ring products); (ii) P_tr_ undergoes •OH-induced cleavage to one-ring P_or_ (k_B_, Equation (4)); and (iii) direct mineralization of P_or_ (k_M_, Equation (5)) was assumed, as aliphatic intermediates were undetected by GC/MS (though implied by organic matter removal (TOC) results). These assumptions are consistent with previous findings that BPA degradation produces numerous intermediates through complex, competing pathways [58,59,60].

The kinetic model resolves the governing relationships of •OH generation and interconversion, where •OH radicals recombine to form H_2_O_2_. Equation (6) represents the fundamental recombination equilibrium between •OH and H_2_O_2_ during anodic water electrolysis [14]. This equilibrium gives rise to Equation (7), which quantifies their competitive formation dynamics. Most significantly, the equilibrium position enables back-calculation of transient •OH concentrations from experimentally measured H_2_O_2_ levels (Equation (8)). Collectively, these equations establish a quantitative framework to characterize the electrode’s operational •OH yield the critical driver of the degradation mechanisms outlined in Equations (3)–(5).

(3) $$BPA+•OH\overset{k_{S}}{\to }P_{tr}$$

(4) $$P_{tr}+•OH\overset{k_{B}}{\to }P_{or}$$

(5) $$P_{or}+•OH\overset{k_{M}}{\to }{CO}_{2}+H_{2}O$$

(6) $$•OH+•OH\begin{array}{c} \overset{k_{1}}{\to }\\ \underset{k_{2}}{\leftarrow } \end{array}H_{2}O_{2}$$

Differential rate laws were applied to describe •OH-involving reactions: BPA depletion (Equation (9)), P_tr_ depletion (Equation (10)), P_or_ depletion (Equation (11)), and CO_2_ evolution (Equation (12)). Equation (13) expresses the mass balance for the system, where [BPA]_0_ represents the initial BPA concentration. This conservation law ensures full carbon accounting throughout the electrooxidation process. As experimental observations confirmed that •OH-mediated oxidation overwhelmingly surpassed adsorptive processes, the adsorption of BPA on the electrode was considered negligible and was not included in the model (Supplementary Materials Text S1 and Table S1). The complete data processing methodology and normalization procedures are detailed in Supplementary Materials (Text S2).

(7) $${k_{1}\cdot \left[•OH\right]}_{t}{\cdot \left[•OH\right]}_{t}=k_{2}\cdot {\left[H_{2}O_{2}\right]}_{t}$$

(8) $${\left[•OH\right]}_{t}=constant\cdot \sqrt{{\left[H_{2}O_{2}\right]}_{t}}$$

(9) $$\frac{d{\left[BPA\right]}_{t}}{dt}=-k_{S}{\cdot {\left[BPA\right]}_{t}\cdot \left[•OH\right]}_{t}$$

(10) $$\frac{d{\left[P_{tr}\right]}_{t}}{dt}=k_{S}{\cdot {\left[BPA\right]}_{t}\cdot \left[•OH\right]}_{t}-k_{B}{\cdot {\left[P_{tr}\right]}_{t}\cdot \left[•OH\right]}_{t}$$

(11) $$\frac{d{\left[P_{or}\right]}_{t}}{dt}=k_{B}{\cdot {\left[P_{tr}\right]}_{t}\cdot \left[•OH\right]}_{t}-k_{M}{\cdot {\left[P_{or}\right]}_{t}\cdot \left[•OH\right]}_{t}$$

(12) $$\frac{d{\left[{CO}_{2}\right]}_{t}}{dt}=k_{M}{\cdot {\left[P_{or}\right]}_{t}\cdot \left[•OH\right]}_{t}$$

(13) $${\left[BPA\right]}_{0}={\left[BPA\right]}_{t}+{\left[P_{tr}\right]}_{t}+{\left[P_{or}\right]}_{t}+{\left[{CO}_{2}\right]}_{t}$$

To ensure robust parameter estimation, we implemented a normalization scheme where concentrations were normalized as [i]* = [i]_t_/[BPA]_0_ (for i = BPA, P_tr_, P_or_, CO_2_, •OH, H_2_O_2_) and time was scaled as t* = *t*/(1∙10^−6^ s) to avoid optimization convergence issues (Equation (14)). The resulting dimensionless equations (Equations (15)–(20)) enabled estimation of four key parameters: the proportionality factor k, and dimensionless rate constants $k_{S}^{*}$, $k_{B}^{*}$ and $k_{M}^{*}$ (constrained to 0–1 range to avoid optimization pitfalls).

(14) $$\frac{d{\left[i\right]}^{*}}{dt^{*}}=\frac{d({\left[i\right]}_{t}/{\left[BPA\right]}_{0})}{d(t/scaling\;factor)}=\frac{scaling\;factor}{{\left[BPA\right]}_{0}}\cdot \frac{d{\left[i\right]}_{t}}{dt}$$

(15) $$\frac{d{\left[BPA\right]}^{*}}{dt^{*}}=-k_{S}^{*}\cdot {\left[BPA\right]}^{*}\cdot {\left[•OH\right]}^{*}$$

(16) $$\frac{d{\left[P_{tr}\right]}^{*}}{dt^{*}}=k_{S}^{*}\cdot {\left[BPA\right]}^{*}\cdot {\left[•OH\right]}^{*}-k_{B}^{*}\cdot {\left[P_{tr}\right]}^{*}\cdot {\left[•OH\right]}^{*}$$

(17) $$\frac{d{\left[P_{or}\right]}^{*}}{dt^{*}}=k_{B}^{*}\cdot {\left[P_{tr}\right]}^{*}\cdot {\left[•OH\right]}^{*}-k_{M}^{*}\cdot {\left[P_{or}\right]}^{*}\cdot {\left[•OH\right]}^{*}$$

(18) $$\frac{d{\left[{CO}_{2}\right]}^{*}}{dt^{*}}=k_{M}^{*}\cdot {\left[P_{or}\right]}^{*}\cdot {\left[•OH\right]}^{*}$$

(19) $${\left[BPA\right]}^{*}+{\left[P_{tr}\right]}^{*}+{\left[P_{or}\right]}^{*}+{\left[{CO}_{2}\right]}^{*}=1$$

(20) $${\left[•OH\right]}^{*}=\frac{constant}{\sqrt{{\left[BPA\right]}_{0}}}\cdot \sqrt{{\left[H_{2}O_{2}\right]}^{*}}=k\cdot \sqrt{{\left[H_{2}O_{2}\right]}^{*}}$$

Absolute rate constants ($k_{S}$, $k_{B}$ and $k_{M}$) were subsequently derived through scaling relationships (Equation (21)). All kinetic parameters were estimated in time units of seconds, consistent with standard rate constant conventions (1/(M∙s)). This normalized kinetic framework provides a comprehensive description of the anodic oxidation process while maintaining numerical stability during parameter optimization.

(21) $$k_{j}=\frac{k_{j}^{*}}{\left(scaling\;factor\cdot {\left[BPA\right]}_{0}\right)}$$

### 2.3. Hydroxyl Radical Concentration Dynamics from H_2_O_2_ Experimental Measurements

**Figure 2:**
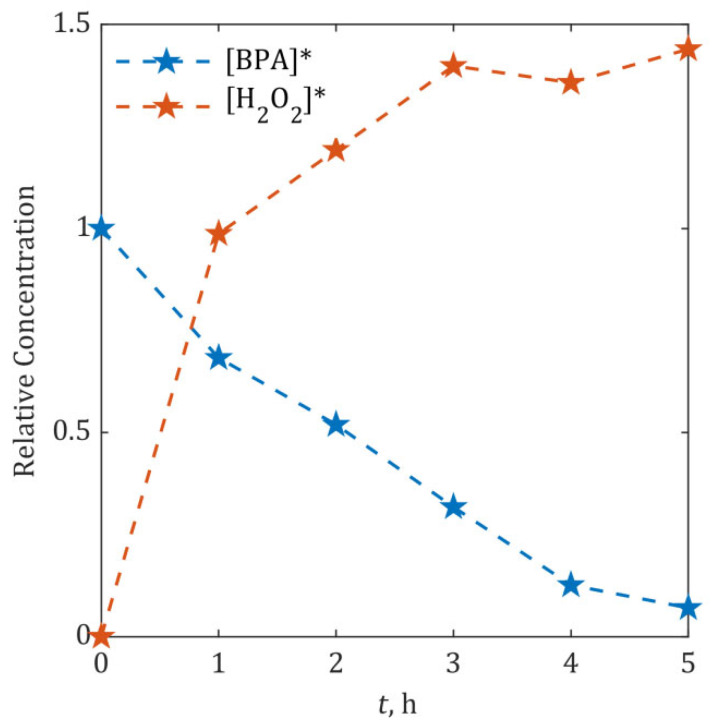
Measured relative BPA and H_2_O_2_ concentrations ([BPA]* and [H_2_O_2_]*) during 5-h anodic oxidation at 15 mA/cm^2^ (SnO_2_-MWCNT@SS anode). Asterisk (*) denotes normalized concentration.

The hydrogen peroxide time evolution measurement was used to calculate hydroxyl radical concentration, according to Equation (20). The relative concentrations of BPA and H_2_O_2_, monitored hourly during 5-h degradation experiments at 15 mA/cm^2^, are represented in Figure 2.

In order to derive the time concentration profile of the •OH radical, the H_2_O_2_ concentration data were fitted by a polynomial. A third-order polynomial (Equation (22)) yielded the optimal fit for the experimental data in Figure 2 (R^2^ = 0.977), with coefficients: $B_{0}=3.09\cdot 10^{-2}$, $B_{1}=3.39\cdot 10^{-4}\;1/s$, $B_{2}=-3.10\cdot 10^{-8}\;{1/s}^{2}$ and $B_{3}=8.81\cdot 10^{-13}\;{1/s}^{3}$. The fitted curve is shown in Figure S1.

(22) $$f\left(t\right)=\sqrt{{\left[H_{2}O_{2}\right]}^{*}}=B_{0}+B_{1}\cdot t+B_{2}\cdot t^{2}+B_{3}\cdot t^{3}\;⟹{\left[•OH\right]}^{*}=k\cdot \left(B_{0}+B_{1}\cdot t+B_{2}\cdot t^{2}+B_{3}\cdot t^{3}\right)$$

**Figure 3:**
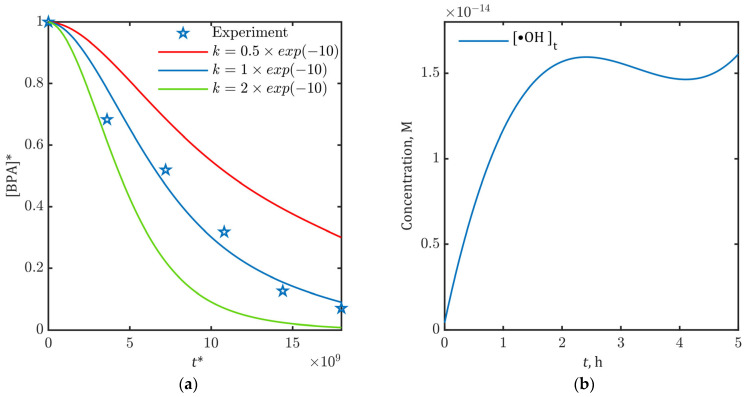
(**a**) Model-predicted relative BPA concentrations (${\left[BPA\right]}^{*}$) for varying proportionality factors (*k*). Asterisk (*) indicates normalized variables; (**b**) Hydroxyl radical (•OH) concentration profile during BPA electrooxidation at 15 mA/cm^2^ (SnO_2_-MWCNT@SS anode).

For initial estimation of the proportionality factor *k*, we used the literature-reported second-order rate constant for BPA reaction with •OH radicals (1∙10^10^ 1/(M∙s)) for all three kinetic constants ($k_{S}$, $k_{B}$ and $k_{M}$) [61,62,63]. The optimal *k* value (1∙10^−10^) was determined by minimizing the discrepancy between model-predicted [BPA]* and experimental data in Figure 2. Results of optimization are represented in Figure 3a. Alternative simulations using *k* values differing by orders of magnitude are provided in Figure S2. This sensitivity analysis (Figure S2) revealed *k* with ±1 order-of-magnitude variation causing >50% deviation in [BPA]* predictions.

The kinetic model enabled the prediction of time-dependent •OH concentrations through Equation (23), using the optimized proportionality factor (*k* = 1 × 10^−10^). Figure 3b presents the simulated •OH profile during BPA electrooxidation at 15 mA/cm^2^, showing characteristic rapid initial generation in the first two hours followed by a quasi-steady-state behavior.

(23) $${\left[•OH\right]}^{*}=k\cdot \left(B_{0}+B_{1}\cdot t^{*}\cdot scaling\;factor+B_{2}\cdot {{(t}^{*}\cdot scaling\;factor)}^{2}+B_{3}\cdot ({t^{*}\cdot scaling\;factor)}^{3}\right)$$

### 2.4. Determination of Second-Order Rate Constants via Kinetic Modelling

The proposed kinetic model (Equations (15)–(20)), incorporating ${\left[•OH\right]}^{*}$ estimated from Equation (23), was fitted to the experimental temporal profiles of BPA and its degradation intermediates (Figure 1) to determine the second-order rate constants ($k_{S}$, $k_{B}$ and $k_{M}$). Parameter estimation yielded the following rate constants (95% confidence intervals): $k_{S}=\left(10.02\pm 1.19\right){\cdot 10}^{9}\;1/(M\cdot s)$ (Equation (3)), $k_{B}=\left(3.92\pm 1.48\right)\cdot 10^{10}\;1/(M\cdot s)$ (Equation (4)) and $k_{M}=(13.87\pm 2.64)\cdot 10^{9}\;1/(M\cdot s)$ (Equation (5)). The model demonstrated robust predictive performance, as evidenced by the overall statistical profile: high goodness-of-fit (R^2^ = 0.980 for BPA degradation efficiency and R^2^ = 0.996 for mineralization efficiency, i.e., CO_2_ formation), low prediction errors (RMSE = 0.039, 3.9% relative error), and minimal residual variance (RSS = 0.0105). A complete statistical profile is provided in Table S3 (Supplementary Materials). The notably reduced determination coefficients for P_tr_ (R^2^ = 0.828) and P_or_ (R^2^ = 0.677) are attributed to the lumping approach used in the kinetic model, which combines intermediates with inherently distinct polarity and oxidation kinetics. This variability is reflected in the estimated 95% confidence interval for the composite rate constant $k_{B}=\left(3.92\pm 1.48\right)\cdot 10^{10}\;1/(M\cdot s)$, confirming that the observed deviations originate from grouping species with differing individual oxidation rates rather than model inadequacy. Consequently, the precision of the prediction of concentrations of intermediates could be further increased by improving the precision of experimental analysis that was used to determine input concentrations of intermediates.

**Figure 4:**
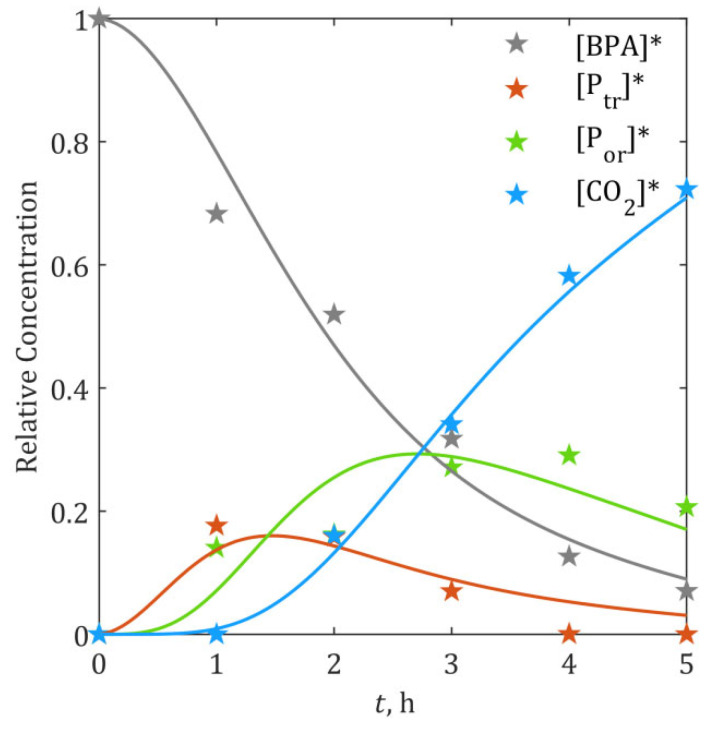
Estimation results of rate constants $k_{S}$, $k_{B}$ and $k_{M}$: ★—experimental data, solid lines—model predictions. Asterisk (*) denotes normalized (relative) concentration.

The close agreement between simulated and experimental concentration profiles (Figure 4) provides strong validation of both the proposed •OH-driven reaction mechanism and the accuracy of parameter estimation methodology, confirming model reliability across the entire 5-h process. Predicted •OH concentrations (Figure 3b) and rate constants also aligned well with literature values for analogous systems [64,65,66], confirming the model’s mechanistic plausibility.

### 2.5. Generalization and Validation of the Model for Different Current Densities

The kinetic model was generalized to predict BPA degradation efficiency across a range of current densities (2.5–20 mA/cm^2^). To establish the current density/rate constant dependence, we incorporated experimental data from Simić et al. [49] while maintaining determined values for the second-order rate constants ($k_{S}=1.002{\cdot 10}^{10}\;1/(M\cdot s)$, $k_{B}=3.920\cdot 10^{10}\;1/(M\cdot s)$, $k_{M}=1.387\cdot 10^{10}\;1/(M\cdot s)$).

**Figure 5:**
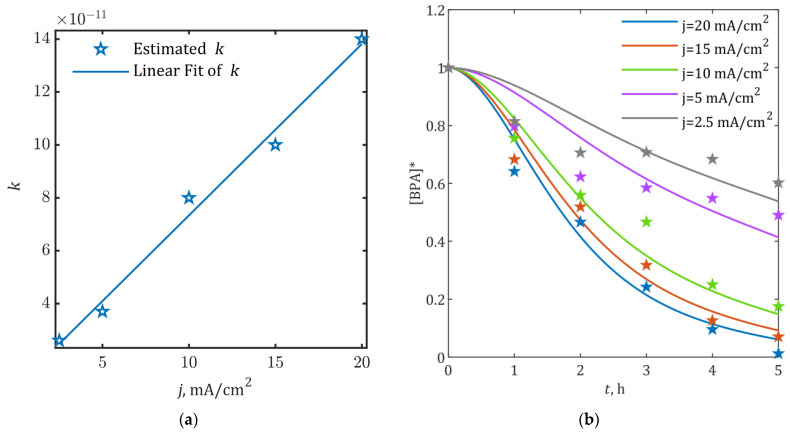
Current-density-dependent model performance: (**a**) Linear correlation between proportionality factor *k* and applied current density *j*; (**b**) Comparison of simulated and experimental [BPA]* profiles across current densities (2.5–20 mA/cm^2^): ★—experimental data, solid lines—model predictions.

The proportionality factor *k*, governing relative •OH concentrations in Equations (20), (22) and (23), exhibited a strong linear correlation with applied current density (R^2^ = 0.989). As shown in Figure 5a, this relationship follows *k*(*j*) = (6.48∙10^−12^ cm^2^/mA)∙*j* + 8.55∙10^−12^, where *j* represents current density. Integration of this linear dependence into the kinetic model yielded simulated [BPA]* profiles that closely matched experimental observations across all tested current densities (Figure 5b).

Model validation was focused on BPA removal efficiency due to the lack of intermediate concentration data. The statistical profile presented in Table 2 demonstrates robust predictive capability, with relative prediction errors (RMSE) below 10% and residual variance (RSS) under 5% across the current density range. Notably, the model showed particularly strong performance at higher current densities (10–20 mA/cm^2^), achieving an average R^2^ ≈ 0.97 with RMSE ≈ 5.66% and RSS ≈ 1.95%. At 15 mA/cm^2^ specifically, the results showed excellent agreement with Figure 4 data, with minimal deviations in determination coefficient (ΔR^2^ = 1.22%) and moderate increases in prediction error (ΔRMSE = 5.37%) and residual variance (ΔRSS = 11.04%).

The predictive potential of the model (R^2^) decreases significantly for lower currents of 2.5 and 5 mA/cm^2^, especially in the first stage of the process, likely due to the inability of low currents to assure irreversible electron transfer. On the other hand, reversibility of the processes (Equations (3)–(5)) was not taken into account in the present model, as the backwards rate constants were neglected for simplicity and clarity.

The model’s maintained accuracy across varying operational conditions confirms its utility for predicting BPA degradation efficiency under different electrochemical treatment scenarios. This current-density-dependent generalized model can serve as both a design tool (predicting *j*-dependent efficiency) and a safety factor (quantified error bounds) for process optimization or potential scale-up applications.

### 2.6. Prediction of Intermediate Concentration and Toxicity Profiles at Different Current Densities

**Figure 6:**
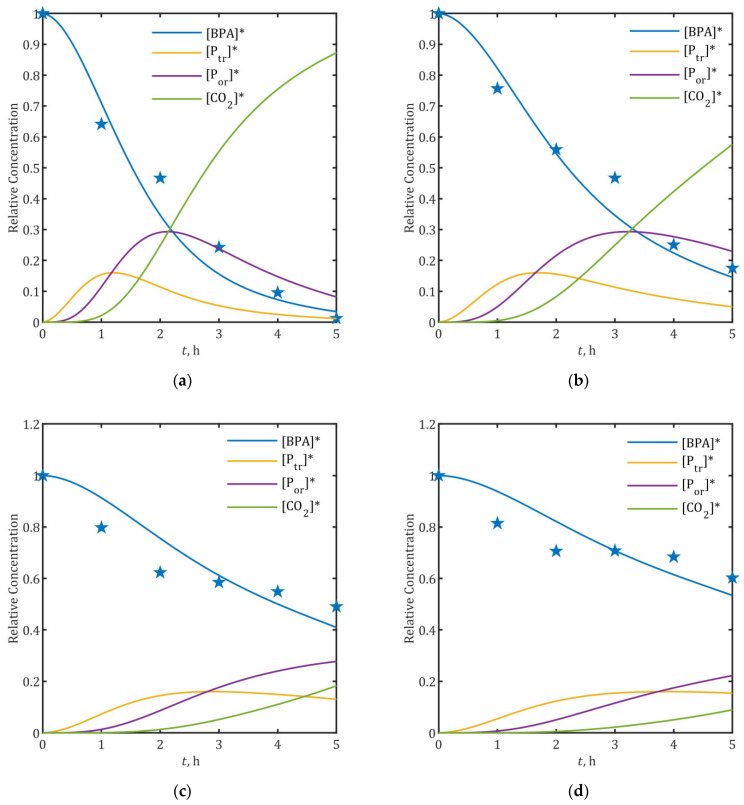
Simulated temporal profiles of BPA*, degradation intermediates (P_tr_*, P_or_*), and CO_2_* during 5-h electrolysis at: (**a**) 20 mA/cm^2^; (**b**) 10 mA/cm^2^; (**c**) 5 mA/cm^2^; and (**d**) 2.5 mA/cm^2^. Experimental data are marked with star symbols (★), while solid lines represent model predictions.

The temporal evolution concentration profile was predicted across current densities (2.5–20 mA/cm^2^) using the kinetic model. Figure 6 presents the simulated concentration profiles of aromatic intermediates and CO_2_, excluding 15 mA/cm^2^ data (previously shown in Figure 3b and Figure 4) for clarity. The corresponding •OH radical concentration profiles are presented in Figure S3.

The model predicts that after 5 h of electrolysis, successful degradation of both two-ring (P_tr_) and one-ring (P_or_) intermediates can only be achieved at 20 mA/cm^2^ (Figure 6a). While 10 mA/cm^2^ treatment effectively degraded more than 90% of P_tr_, P_or_ degradation remained negligible (Figure 6b). Lower current densities (≤5 mA/cm^2^) showed limited treatment efficiency, with less than 60% BPA removal, negligible P_tr_ degradation (<1%), and net accumulation of P_or_ accompanied by low mineralization (≤20%).

**Figure 7:**
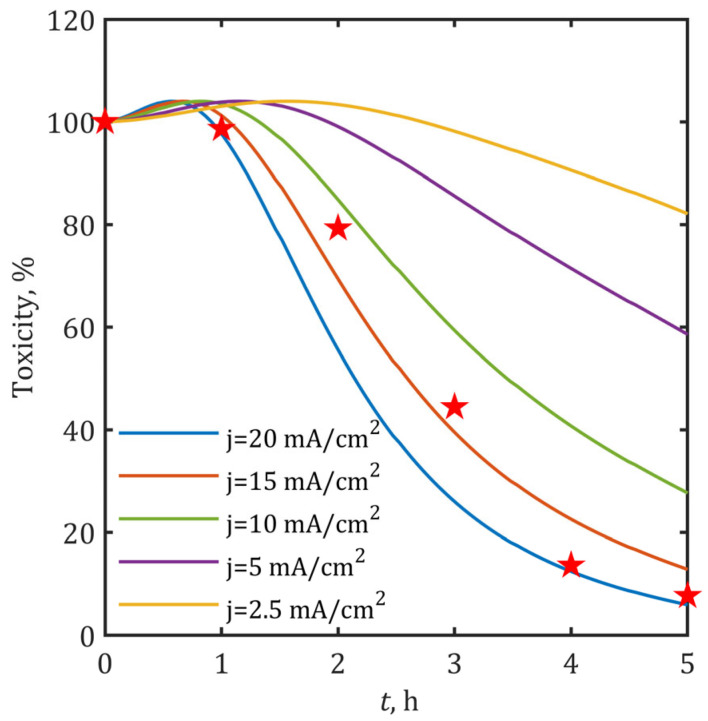
Current-density-dependent model predictions for SnO_2_-MWCNT@SS anode performance: normalized toxicity evolution (%) of treated solutions across applied current densities (2.5–20 mA/cm^2^) during 5-h BPA electrooxidation. Experimental data are marked with star symbols (★), while solid lines represent model predictions.

The developed model successfully predicted temporal toxicity trends in the investigated system, with validation performed against experimental data at 15 mA/cm^2^. As illustrated in Figure 7, the simulated toxicity profile shows strong agreement with experimental results, achieving a determination coefficient of R^2^ = 0.971.

Predicted toxicity trends (Figure 7) reveal critical process dynamics. Toxicity evolution exhibited three distinct phases: (i) an initial 1–3% increase during the first 1–2 h, (ii) a maximum toxicity point, followed by (iii) a rapid decrease. This transition occurred earlier at higher current densities, completing after 1 h at 20 mA/cm^2^ compared to 2 h at 2.5 mA/cm^2^. The rate of toxicity reduction scales with energy input, requiring > 10 mA/cm^2^ to achieve > 80% toxicity reduction within 5 h. These results demonstrate that while partial BPA removal occurs at lower current densities, effective toxicity control requires sufficient •OH generation rates only achieved at ≥10 mA/cm^2^.

This biphasic toxicity profile aligns with the proposed reaction mechanism, where initial oxidation and formation of two-ring products generate more toxic fragments before their degradation to less harmful one-ring products and eventual mineralization up to CO_2_ and water [37]. The findings underscore the importance of optimizing treatment parameters (current density and duration) to navigate the transient toxicity window while achieving maximal detoxification.

### 2.7. Generalizability Assessment for •OH-Mediated Electrochemical Oxidation of Organic Pollutants

The model operates on the premise that •OH radicals serve as the dominant reactive species for BPA degradation, an assumption supported by our earlier radical quenching experiments [49] and literature evidence confirming •OH-mediated aromatic ring attack as the primary pathway [50,51,67]. To assess the model’s broader utility, we applied it to rhodamine B degradation [48], using experimental data obtained with the specific SnO_2_-MWCNT@SS anode system (initial concentration 1.04∙10^−4^ M, 50 ppm; pH ≈ 7; current density 20 mA/cm^2^ over 3 h). UV-Vis spectroscopy served as the analytical method for rhodamine B quantification.

The •OH generation capacity of the electrode, characterized in Figure 5a, yielded a proportionality factor *k* = 1.38∙10^−10^. The degradation pathway for rhodamine B was constructed based on identified intermediates and the mechanism proposed by Dai et al. [68], beginning with cleavage of the conjugated structure to form one-ring mono- and di-carboxylic acids (benzoic acid and its derivatives lumped as P_1_) and followed by aromatic ring opening to produce aliphatic acids (succinic acid and 2-hydroxyglutaric acid lumped as P_2_) that are mineralized to CO_2_ and H_2_O. Kinetic parameters were assigned with k_s_ = $9{\cdot 10}^{9}\;1/(M\cdot s)$ for the initial rhodamine B oxidation [69,70,71] and $k_{B}$ = $k_{M}$ = $1.2{\cdot 10}^{10}\;1/(M\cdot s)$ for subsequent intermediate oxidation [72,73], based on literature values for structurally similar compounds (salicylic acid).

**Figure 8:**
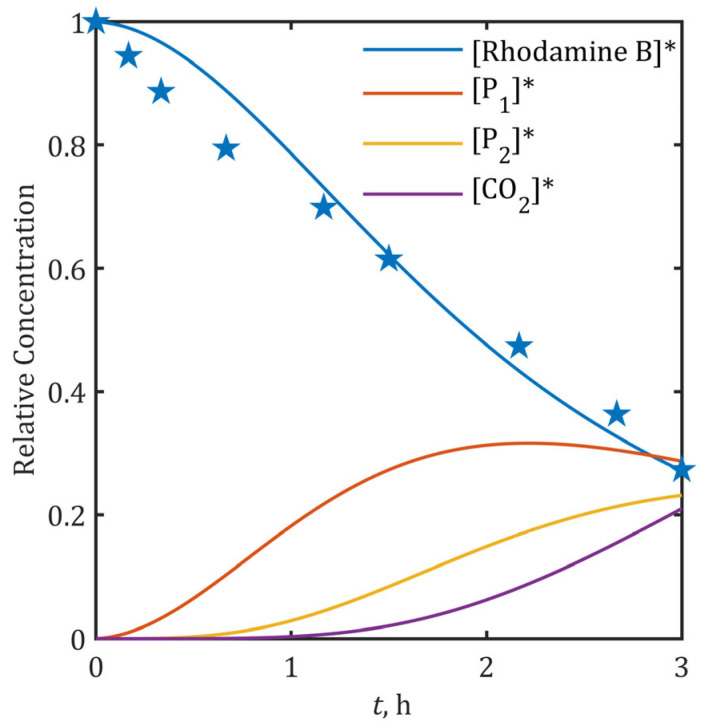
Experimental vs. predicted rhodamine B degradation kinetics at 20 mA/cm^2^. Experimental data are marked with star symbols (★), while solid lines represent model predictions. Asterisk (*) denotes normalized (relative) concentration.

Model predictions demonstrated strong correlation with experimental data (R^2^ = 0.961), as shown in Figure 8, though minor discrepancies during the initial hour suggest the possible existence of transient alternative degradation pathways that become negligible as mineralization progresses. The predicted 20% mineralization efficiency aligns with established literature showing that while decolorization may be extensive, organic matter removal (TOC) often remains below 40% [74,75]. These results validate the model’s reliability in predicting both degradation kinetics of principal pollutants and mineralization efficiency across operationally relevant conditions (different classes of organic pollutants; initial concentrations: 30–50 ppm; pH: 4–7; *j* > 10 mA/cm^2^), despite its simplified reaction framework (Supplementary Materials Table S3). Given that short-duration electrooxidation (0–1 h) yields insufficient degradation/mineralization (<30%, Figure 4, Figure 6 and Figure 8) while generating more toxic intermediates than the parent pollutants (Figure 7), the model’s predictive capability for longer treatment times—when process efficiency becomes practically significant —remains fully valid and operationally relevant.

## 3. Discussion

The concentration of •OH radicals was estimated using equilibrium conditions and an easily measurable concentration of H_2_O_2_, one of the competitive water electrolysis products. The presented semiempirical methodology enables quantification and prediction of •OH radical concentration, the key electrochemical variable in anodic oxidation experiments, being difficult to trace directly [9]. Moreover, when the H_2_O_2_ measuring experiment is once performed, it enables insight into •OH-radical concentration and the predictive estimation of the whole concentration profile of one-ring and two-ring products, even in the absence of GC/MS detection. The results show that once the proportionality factor between •OH radical and H_2_O_2_ concentrations is defined (Equation (20)), the model successfully predicts the concentration distribution of the pollutant based on its initial concentration and second-order rate constant towards •OH radicals. The proposed strategy enables the prediction of the complete electrooxidation process outcome, including degradation efficiency extent and toxicity time profile, using only simple data obtained from H_2_O_2_ measurements within anodic oxidation experiments and available toxicity databases.

The presented estimation procedure includes assessment of the oxidative breakdown of BPA, identifying its key intermediates, and projecting the degradation pathway over a five-hour period of electrochemical oxidation. Based on these results, two crucial steps in the degradation process were identified. The first involves the oxidation of the BPA aliphatic backbone without breaking the chain, while the second entails the direct cleavage of the BPA molecule, yielding one-ring products. Ultimately, the process is proposed to result in the opening of rings and production of diverse aliphatic molecules, which are further oxidized to CO_2_ and H_2_O, as corroborated by the obtained high degrees of complete mineralization.

The model was verified against previously published data on BPA degradation at different current densities. The validated linear relationship between •OH availability and applied current density (Figure 5a) enables predictive control of degradation efficiency across operational scales, while the robust error margins (Table 2) establish reliability boundaries for engineering implementation. The procedure provided a correlation between process efficiency and energy input, emphasizing the uniformity and applicability of the model, at least at the current densities higher than 10 mA/cm^2^. These currents enable satisfactory energy input for irreversible electron transfer, so the reversibility of the oxidation reactions can be neglected.

This current-density-dependent generalization provides both fundamental insights and practical tools for (1) optimizing electrooxidation conditions and (2) facilitating potential scale-up applications. The developed kinetic model can predict the concentration profiles of •OH radicals, the principal pollutant, and its primary intermediate and product. It can also be used to assess process operational parameters such as treatment duration and applied current density, with the purpose of optimization of energy consumption. In connection with the computational databases on the toxicity, as demonstrated in the case of T.E.S.T. (Toxicity Estimation Software Tool), it can be used to estimate the toxicity of the degradation mixture and determine optimal operational conditions for the degradation to minimize toxicity, once all potentially toxic degradation by-products are known. As corroborated by obtained correlation coefficients, the model is particularly applicable for predicting the concentration profile of initial pollutants, the mineralization extents, and the relative toxicity. The prediction of the formation and depletion of intermediates in the tracked system was primarily qualitative, however, with the clear perspective for further improvement of precision. Through comprehensive characterization of oxidative intermediates, the lumping-based classification scheme can be systematically refined to enable precise toxicity predictions from simulated concentration profiles, as demonstrated by Ferreiro et al. in their study of UV/H_2_O_2_-mediated 4-chlorophenol degradation [37].

Finally, the results suggest that the model, which has a principally general character regarding chemical properties of involved species (Figure 8), can be widened to include other reactive species, if necessary more specifically defined. This would allow more complex kinetic studies of anodic oxidation or related H_2_O_2_ mediated EAOPs processes [35].

## 4. Materials and Methods

### 4.1. Electrochemical Oxidation

The SnO_2_-MWCNT@SS anode modeled in this study was prepared as described in our prior work [49], with essential procedural details summarized below. The SnO_2_/MWCNT nanocomposite was synthesized by dispersing SnO_2_ nanoparticles with functionalized multi-walled carbon nanotubes (MWCNTs, dimensions: 7–15 nm × 3–6 nm × 0.5–200 µm; Sigma-Aldrich, St. Louis, MO, USA) in dimethylformamide (DMF, Sigma-Aldrich, St. Louis, MO, USA) at a 3.5:1 (*w*/*w*) ratio. The mixture was sonicated for 5 h at room temperature to achieve a homogeneous suspension (4.5 mg/mL). A total of ~210 µL suspension (70 µL per application) was drop-cast onto a stainless steel substrate (1 × 2 cm, 2 cm^2^ surface area) in three sequential layers, with each layer dried for 20 min under a 250 W infrared lamp. As validated in [49], structural and electrochemical characterization confirmed uniform nanocomposite distribution, stable anode performance, and enhanced electroactive surface area.

Bisphenol A (BPA, Sigma-Aldrich, St. Louis, MO, USA) degradation was investigated in a two-electrode electrochemical cell containing 60 mL of 0.1 M Na_2_SO_4_ electrolyte with 1.31∙10^−4^ M BPA (30 ppm, pH ≈ 4). The system utilized an SnO_2_-MWCNT@SS working electrode and a stainless steel counter electrode. Chronopotentiometric experiments were performed for 5 h using a Gamry Interface 1000 Potentiostat/Galvanostat (Gamry Instruments, Warminster, PA, USA), with 0.5 mL aliquots collected at 1-h intervals for subsequent analysis. While reference [49] reported BPA degradation across multiple current densities (2.5–20 mA/cm^2^), this study focused exclusively on 15 mA/cm^2^ for mechanistic analysis.

Aliquots were subjected to two parallel analytical workflows. GC-MS analysis (Agilent 7890B/5977A, Agilent Technologies, Inc., Santa Clara, CA, USA) enabled quantification of BPA degradation kinetics and identification of reaction intermediates. The measurement descriptions relevant to this study are provided in Text S3 (Supplementary Materials). Simultaneously, UV-Vis spectroscopy (Lambda 35, Perkin Elmer, Waltham, MA, USA) monitored H_2_O_2_ formation via the titanium oxalate method [76], capturing its competitive generation alongside •OH radicals during water electrolysis. This dual-method approach provided comprehensive insight into both organic pollutant degradation and oxidant production dynamics. Total organic carbon (TOC) measurements were performed using a TOC-LCPH analyzer (Shimadzu Co., Kyoto, Japan) to quantify mineralization efficiency through the reduction in organic carbon content during electrooxidation.

### 4.2. Time-Dependent Toxicity Profiling During BPA Electrooxidation

Acute toxicity of BPA and its degradation intermediates was evaluated using the United States Environmental Protection Agency’s (U.S. EPA’s) Toxicity Estimation Software Tool (T.E.S.T., version 5.1.2) with the Consensus quantitative structure-activity relationship (QSAR) model. Toxicity was expressed as LC_50_ values, representing the aqueous concentration (mg/L) causing 50% mortality in Fathead Minnow (*Pimephales promelas*) after 96-h exposure.

The temporal toxicity profile was derived from the relative acute toxicities of BPA and its pseudo-intermediates (two-ring derivatives P_tr_ and one-ring derivatives P_or_), calculated as

(24) $$Toxicity\left(\%\right)=100\cdot \frac{{{LC}_{50}}_{BPA}}{M_{BPA}}\cdot \sum_{i}\frac{{\left[i\right]}^{*}}{\;{{LC}_{50}}_{i}/M_{i}}=100\cdot {LC}_{50_{BPA}}^{*}\cdot \sum_{i}\frac{{\left[i\right]}^{*}}{{LC}_{50_{i}}^{*}}$$

where i denotes BPA, P_tr_ and P_or_, and ${{LC}_{50}}_{BPA}$ is the reference toxicity value. LC_50i_ represents the acute toxicity of individual components (BPA, P_tr_ and P_or_), and M_i_ denotes their corresponding molar masses. The full derivation of these relationships is provided in Supplementary Materials (Text S4).

### 4.3. Mathematical Modelling

Model parameters were estimated using experimental data from BPA electrooxidation at 15 mA/cm^2^ with the SnO_2_-MWCNT@SS anode, including temporal profiles of relative concentrations for BPA, degradation intermediates (P_tr_ and P_or_), H_2_O_2_, and CO_2_. For validation, independent datasets of BPA degradation at additional current densities (5–20 mA/cm^2^) from [49] were employed.

Parameter optimization was implemented in MATLAB^®^ (The MathWorks, Inc., Natick, MA, USA [77]) via the Optimization Toolbox™ lsqcurvefit function [78], utilizing a trust-region-reflective algorithm to solve the nonlinear least-squares problem [79,80]. Process simulations were performed using the ode45 solver [81], which implements an explicit Runge-Kutta fourth- and fifth-order method for nonstiff differential equations [82,83].

## Figures and Tables

**Table 1:** Organic compounds identified by gas chromatography/mass spectrometry (GC/MS) during 5 h of bisphenol A (BPA) electrooxidation using a tin oxide-modified multi-walled carbon nanotubes on stainless steel anode (SnO_2_-MWCNT@SS).

Compound Name	Structural Formula [56]	Retention Time (min)	Molar Mass (g/mol)	96-h LC_50_ Fathead Minnow (mg/L)
Bisphenol A C_15_H_16_O_2_	** 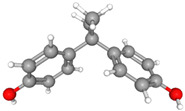 **	23.15	228.29	3.24
Product A 2-(3,4-dihydroxyphenyl)-2-(4-hydroxyphenyl)acetaldehyde C_14_H_12_O_4_	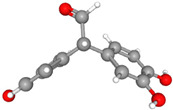	24.33	244.24	1.83
Product B 2-(2-4-dihydroxyphenyl)-2-(4-hydroxyphenyl)acetaldehyde C_14_H_12_O_4_	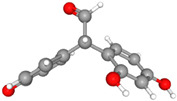	24.05	244.24	2.26
4-hydroxybenzoic acid C_7_H_6_O_3_	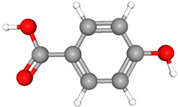	16.5	138.12	92.62
Hydroquinone C_6_H_6_O_2_	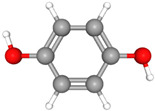	13.45	110.11	43.29
4-isopropenylphenol C_9_H_16_O	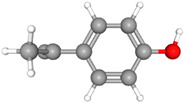	12.9	140.22	7.67
Benzoic Acid C_7_H_6_O_2_	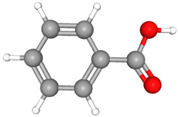	11.1	122.12	101.5

**Table 2:** Statistical evaluation of current-density-dependent model predictions for BPA removal efficiency. Metrics include determination coefficient (R^2^), root mean square error (RMSE), and residual sum of squares (RSS) for each tested current density (*j*).

*j*, mA/cm^2^	R^2^	RMSE, 1	RSS, 1
20	0.9623	0.0659	0.0260
15	0.9712	0.0549	0.0181
10	0.9700	0.0491	0.0144
5	0.7536	0.0863	0.0447
2.5	0.6157	0.0786	0.0371

## Data Availability

The original contributions presented in this study are included in the article/Supplementary Materials. Further inquiries can be directed to the corresponding authors.

## Supplementary Materials

The following supporting information can be downloaded at: https://www.mdpi.com/article/10.3390/ijms26104785/s1.

## Abbreviations

The following abbreviations are used in this manuscript.

	Letter designations
AOPs	Advanced oxidation processes
BPA	Bisphenol A
EAOPs	Electrochemical advanced oxidation processes
EF	Electro-Fenton process
EO	Electrooxidation process
EPR	Electron paramagnetic resonance spectroscopy
HO_2_•	Hydroperoxyl radicals
•OH	Hydroxyl radical
P_or_	Lumped one-ring BPA degradation intermediates
P_tr_	Lumped two-ring BPA degradation intermediates
P_1_	Lumped aromatic rhodamine B degradation intermediates
P_2_	Lumped aliphatic rhodamine B degradation intermediates
SnO_2_-MWCNT@SS	Tin oxide/multi-walled carbon nanotube composite on stainless steel substrate
SO_4_•^−^	Sulfate radical
T.E.S.T.	U.S. EPA’s toxicity estimation software tool
	Physical Quantities
[BPA]_0_	Initial BPA concentration (M)
$B_{0}$	Zero-order polynomial coefficient for H_2_O_2_ fit (dimensionless)
$B_{1}$	First-order polynomial coefficient for H_2_O_2_ fit (1/s)
$B_{2}$	Second-order polynomial coefficient for H_2_O_2_ fit (1/s^2^)
$B_{3}$	Third-order polynomial coefficient for H_2_O_2_ fit (1/s^3^)
[i]_t_	Time-dependent concentration of species i = BPA, P_tr_, P_or_, CO_2_, •OH, H_2_O_2_ (M)
[i]*	Relative concentration of species i = BPA, P_tr_, P_or_, CO_2_, •OH, H_2_O_2_ (dimensionless)
*j*	Current density (mA/cm^2^)
k	Proportionality factor (dimensionless)
$k_{•OH}^{pollutant}$	Pseudo-second-order rate constant of •OH radicals with pollutants (1/(M∙s))
k_B_	Second-order rate constant for P_tr_ cleavage (1/(M∙s))
k_M_	Second-order rate constant for P_or_ mineralization (1/(M∙s))
k_S_	Second-order rate constant for BPA degradation (1/(M∙s))
$k_{B}^{*}$	Normalized rate constant for P_tr_ cleavage (dimensionless)
$k_{M}^{*}$	Normalized rate constant for P_or_ mineralization (dimensionless)
$k_{S}^{*}$	Normalized rate constant for BPA degradation (dimensionless)
LC_50_	96-h acute toxicity for *Pimephales promelas* (Fathead Minnow) (mg/L)
M	Molar mass (g/mol)
R^2^	Coefficient of determination (dimensionless)
RMSE	Root mean square error (units match predicted variable)
RSS	Residual sum of squares (matches the square of the predicted variable’s units)
*t*	Time (s)
*t**	Scaled time (dimensionless)
TOC	Total organic carbon content (mg/L)
V	System volume (L)
	Mathematical Operators
∆	Difference operator
∑	Summation operator
