# Supplementary material for: Predicting the Fate of Bisphenol A During Electrochemical Oxidation: A Simple Semiempirical Method Based on the Concentration Profile of Hydroxyl Radicals
Source: Int J Mol Sci. 2025 May 16;26(10):4785. doi: 10.3390/ijms26104785 (PMC12112118; doi:10.3390/ijms26104785)
Supplement: Supplementary file 1 [file ijms-26-04785-s001.zip › ijms-3618322-supplementary.pdf]

Supplementary Materials for

Predicting the fate of BPA during electrochemical oxidation: a simple semiempirical method based on the concentration profile of hydroxyl radicals

Marija Ječmenica Dučić [1,*], Dragana Vasić Anićijević [1], Danka Aćimović [1], Ľubomír Švorc [2], Branko Bugarski [3], Radojica Pešić [3], and Tanja Brdarić [1]

[1] University of Belgrade, VINČA Institute of Nuclear Sciences-National Institute of the Republic of Serbia, Department of Physical Chemistry, Mike Petrovića Alasa 12-14, 11000, Belgrade, Serbia; draganav@vin.bg.ac.rs; dankavla@vin.bg.ac.rs; tanja.brdaric@vin.bg.ac.rs

[2] Slovak University of Technology in Bratislava, Institute of Analytical Chemistry, Faculty of Chemical and Food Technology, Radlinského 9, 812 37 Bratislava, Slovakia; lubomir.svorc@stuba.sk

[3] University of Belgrade, Faculty of Technology and Metallurgy, Department of Chemical Engineering, Karnegijeva 4, 11000, Belgrade, Serbia; branko@tmf.bg.ac.rs; rpesic@tmf.bg.ac.rs

*** Corresponding author**

Marija Ječmenica Dučić

University of Belgrade

VINČA Institute of Nuclear Sciences-National Institute of the Republic of Serbia

Department of Physical Chemistry

Mike Petrovića Alasa 12-14

Belgrade, 11000

marija.jecmenica@vin.bg.ac.rs

Text S1. *TOC analysis and pseudo-intermediate quantification*

The model assumes isothermal (25 °C) and isobaric (1 atm) conditions with an initial system volume (V_0) of 60 mL. Volume changes throughout the electrooxidation process (from CO_2 evolution) were quantified as: $\Delta V/V_0$ = 0.04 % at 2.5 mA/cm², $\Delta V/V_0$ = 0.11 % at 5 mA/cm², $\Delta V/V_0$ = 0.26 % at 10 mA/cm², $\Delta V/V_0$ = 0.36 % at 15 mA/cm², $\Delta V/V_0$ = 0.42 % at 20 mA/cm². Given these negligible variations (< 0.5 % of V_0 across all current densities), the constant-volume approximation remains valid for kinetic modelling.

The temporal evolution of BPA, its degradation pseudo-intermediates (P_{tr}, P_{or}), and CO_2 is governed by the carbon mass balance equation:

$$[\mathrm{BPA}]^* + [\mathrm{P_{tr}}]^* + [\mathrm{P_{or}}]^* + [\mathrm{CO_2}]^* = 1 \qquad \text{(S1)}$$

Temporal concentrations of BPA ($[BPA]_t$) were determined using GC/MS calibration curves from standard solutions. CO_2 evolution was equated to mineralization efficiency, calculated from the relative decrease in total organic carbon (TOC) abundance during electrooxidation. This relationship is expressed as:

$$\text{Mineralization (\%)} = \left(1 - \frac{\mathrm{TOC}_t}{\mathrm{TOC}_0}\right) \cdot 100 \qquad \text{(S2)}$$

where $[TOC]_0$ and $[TOC]_t$ represent initial and time-dependent TOC values, respectively. The derived CO_2 profile (Table S1) served as a direct metric for complete oxidation to inorganic carbon.

Table S1. Temporal evolution of mineralization efficiency for electrooxidation of BPA at 15 mA/cm² using SnO_2-MWCNT@SS anode

Time, h	Mineralization, %	$[CO_2]^*$
0	0	0
1	0.55	0.0055
2	9.38	0.0938
3	41.2	0.412
4	66.2	0.662
5	84.0	0.84

Collective temporal evolution of BPA and CO_2 is expressed as:

$$\sum\nolimits_1 \mathrm{S} = [\mathrm{BPA}]^* + [\mathrm{CO_2}]^* \qquad \text{(S3)}$$

Peak areas of individual intermediates (Product A, Product B, 4-hydroxybenzoic acid, hydroquinone, 4-isopropenylphenol, benzoic acid) were normalized to the maximum observed value during electrooxidation at 15 mA/cm²:

$$\mathrm{Max} = \mathit{Maximum}(\text{Peak area}(t)) \qquad \text{(S4)}$$

$$\mathrm{I_i}(t) = \frac{\text{Peak area}_\mathrm{i}(t)}{\mathrm{Max}} \qquad \text{(S5)}$$

Their collective abundance was defined as:

$$\sum\nolimits_2 S = \sum_{i}^{n} I_i(t) \tag{S6}$$

The relative concentration of each intermediate was computed as:

$$[i]^* = I_i(t) \cdot \frac{(1 - \sum_1 S)}{\sum_2 S} \tag{S7}$$

Pseudo-intermediate profiles were then aggregated: two-ring derivatives (P_{tr}) – sum of Product A and Product B and one-ring derivatives (P_{or}) – sum of 4-hydroxybenzoic acid, hydroquinone, 4-isopropenylphenol, and benzoic acid:

$$[P_{tr}]^* = \sum_{j}^{n} [j]^* \tag{S8}$$

$$[P_{or}]^* = \sum_{k}^{n} [k]^* \tag{S9}$$

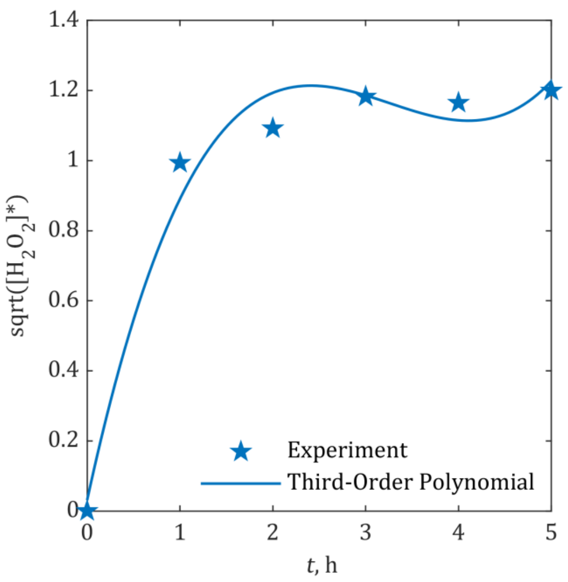

Figure S1. Third-order polynomial fit ($R^2 = 0.977$) of the $\sqrt{[H_2O_2]^*}$ versus time relationship during BPA electrooxidation at 15 mA/cm² (SnO_2-MWCNT@SS anode).

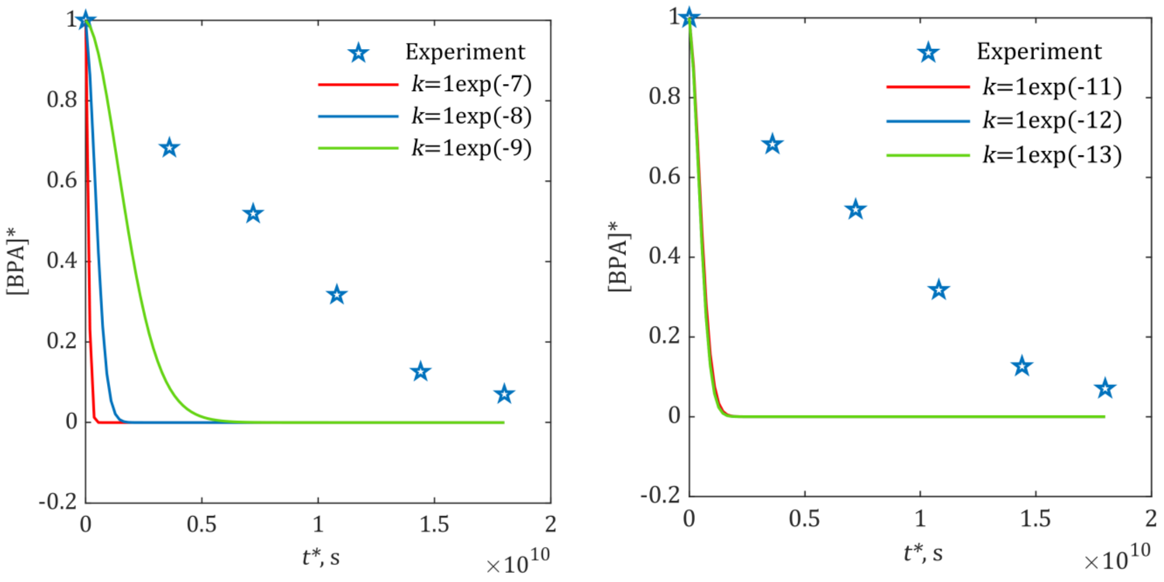

(**a**) (**b**)

Figure S2. Sensitivity analysis of BPA degradation kinetics to the proportionality factor k: Predictions with (**a**) k higher than $1 \cdot 10^{-10}$ and (**b**) k lower than $1 \cdot 10^{-10}$. [BPA]* represents normalized BPA concentration, with solid lines showing model predictions and symbols indicating experimental data at 15 mA/cm^2.

Table S2. Statistical evaluation of kinetic model performance: root mean square error (RMSE) and residual sum of squares (RSS) values for all reactive species in the BPA degradation (BPA, P_{tr}, P_{or}, and CO_2).

**Reactive species**	**RMSE, 1**	**RSS, 1**
[BPA*]	0.0521	0.0163
[P_{tr}^*]	0.0311	0.0058
[P_{or}^*]	0.0547	0.018
[CO_2^*]	0.0181	0.002
Average	0.039	0.010525

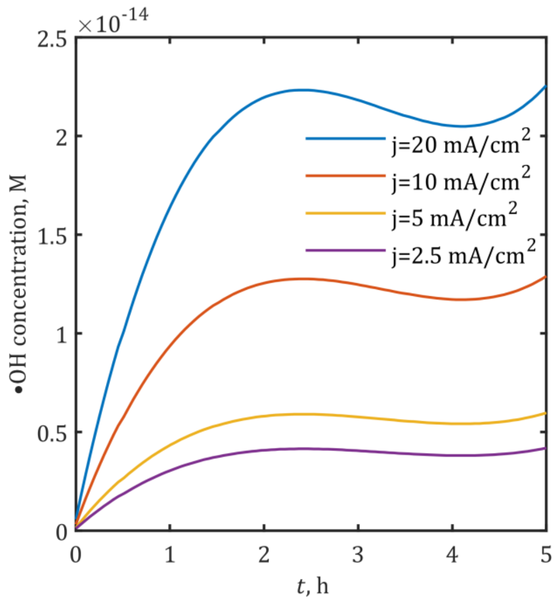

Figure S3. Current-density-dependent model predictions: Hydroxyl radical concentration profiles in treated solutions during 5-hour electrooxidation across current densities (2.5–20 mA/cm^2) for BPA electrooxidation using SnO_2-MWCNT@SS anode

Text S2. *GC/MS analysis*

BPA and its degradation intermediates were extracted using modified liquid-liquid extraction methods adapted from literature protocols [4],[5]. Prior to extraction, sample pH was adjusted to 2.0 using hydrochloric acid (1 M) to ensure proper analyte protonation. Solid sodium chloride was added to promote salting-out effects, thereby enhancing organic phase partitioning. Extraction was performed using 3 mL of diethyl ether with vigorous shaking and repeated three times, yielding a total organic phase volume of 9 mL. The resulting sample was dried with anhydrous sodium sulphate and filtered through a PTFE membrane filter to remove residual particulates. The extract was then concentrated to 0.5 mL under a gentle nitrogen stream and derivatized with 20 µL BSTFA in 50 µL acetone at 70 °C for 30 minutes to improve chromatographic resolution of polar intermediates.

Analyses were performed using an Agilent 7890B GC system coupled with a mass spectrometer (Agilent Technologies, USA), equipped with an HP-5ms capillary column (30 m × 0.25 mm ID, 0.25 µm film thickness). Helium carrier gas (grade 5.0) flowed at 1.0 mL/min. The temperature program initiated at 150 °C (0.5 min hold), ramped at 20 °C/min to 280 °C (5 min hold), then at 40 °C/min to 300 °C (2.5 min hold), with a 250 °C injector and 300 °C transfer line.

For enhanced structural identification, selected samples were analysed via direct infusion mass spectrometry (Waters Micromass Quattro micro API TQD) in negative ion mode (50–500 Da). Optimized parameters included: 3.67 kV capillary voltage, 47 V cone voltage, 120 °C source temperature, and 350 °C desolvation temperature with 500 L/h nitrogen flow. Samples were diluted 1:1 with deionized water prior to MS analysis.

Text S3. *Toxicity analysis*

All data used in toxicity calculations are presented in Table 1 (Main Manuscript). The acute toxicity of BPA was defined by its experimental LC_{50} value of 3.24 mg/L. For degradation

intermediates, toxicity parameters and molar masses were calculated as arithmetic means of constituent compounds:

$$\mathrm{LC}_{50_{\mathrm{P_{tr}}}} = \frac{1}{n} \cdot \sum_{\mathrm{i}}^{n} \mathrm{LC}_{50_{\mathrm{i}}} = 2.045\ \mathrm{mg/L} \tag{S10}$$

$$\mathrm{M}_{\mathrm{P_{tr}}} = \frac{1}{n} \cdot \sum_{\mathrm{i}}^{n} \mathrm{M}_{\mathrm{i}} = 244.24\ \mathrm{g/mol} \tag{S11}$$

where i = Product A and Product B; and

$$\mathrm{LC}_{50_{\mathrm{P_{or}}}} = \frac{1}{n} \cdot \sum_{\mathrm{j}}^{n} \mathrm{LC}_{50_{\mathrm{j}}} = 61.27\ \mathrm{mg/L} \tag{S12}$$

$$\mathrm{M}_{\mathrm{P_{or}}} = \frac{1}{n} \cdot \sum_{\mathrm{j}}^{n} \mathrm{M}_{\mathrm{j}} = 127.6425\ \mathrm{g/mol} \tag{S13}$$

where j = 4-hydroxybenzoic acid, hydroquinone, 4-isopropenylphenol and benzoic acid.

The initial system toxicity was calculated as:

$$\mathrm{Toxicity} = \frac{[\mathrm{BPA}]_0}{\mathrm{LC}^*_{50_{\mathrm{BPA}}}} \tag{S14}$$

with temporal evolution described by Equation S15:

$$\mathrm{Toxicity}\ (t) = \frac{[\mathrm{BPA}]_{\mathrm{t}}}{\mathrm{LC}^*_{50_{\mathrm{BPA}}}} + \frac{[\mathrm{P_{tr}}]_{\mathrm{t}}}{\mathrm{LC}^*_{50_{\mathrm{P_{tr}}}}} + \frac{[\mathrm{P_{or}}]_{\mathrm{t}}}{\mathrm{LC}^*_{50_{\mathrm{P_{or}}}}} = \sum_{\mathrm{i}} \frac{[\mathrm{i}]_{\mathrm{t}}}{\mathrm{LC}^*_{50_{\mathrm{i}}}} \tag{S15}$$

where i = BPA, P_{tr}, P_{or}, and:

$$\mathrm{LC}^*_{50_{\mathrm{i}}} = \frac{\mathrm{LC}_{50_{\mathrm{i}}}}{\mathrm{M}_{\mathrm{i}}} \tag{S16}$$

Normalizing to initial conditions yields the relative toxicity percentage (Equation 24, Main Manuscript):

$$\mathrm{Toxicity}\ (\%) = 100 \cdot \frac{\mathrm{LC}_{50_{\mathrm{BPA}}}}{[\mathrm{BPA}]_0} \sum_{\mathrm{i}} \frac{[\mathrm{i}]_{\mathrm{t}}}{\mathrm{LC}^*_{50_{\mathrm{i}}}} = 100 \cdot \mathrm{LC}^*_{50_{\mathrm{BPA}}} \cdot \sum_{\mathrm{i}} \frac{[\mathrm{i}]^*}{\mathrm{LC}^*_{50_{\mathrm{i}}}} \tag{S17}$$

Under constant volume assumption (V = const.), the average LC_{50} of treated solutions was derived from the mass balance of toxic components (i = BPA, P_{tr}, P_{or}):

$$\frac{\mathrm{M_{BPA}} \cdot [\mathrm{BPA}]^*}{\mathrm{LC}_{50_{\mathrm{BPA}}}} + \frac{\mathrm{M}_{\mathrm{P_{tr}}} \cdot [\mathrm{P_{tr}}]^*}{\mathrm{LC}_{50_{\mathrm{P_{tr}}}}} + \frac{\mathrm{M}_{\mathrm{P_{or}}} \cdot [\mathrm{P_{or}}]^*}{\mathrm{LC}_{50_{\mathrm{P_{or}}}}} = \frac{\mathrm{M_{BPA}} \cdot [\mathrm{BPA}]^* + \mathrm{M}_{\mathrm{P_{tr}}} \cdot [\mathrm{P_{tr}}]^* + \mathrm{M}_{\mathrm{P_{or}}} \cdot [\mathrm{P_{or}}]^*}{\mathrm{LC}_{50}} \tag{S18}$$

Rearrangement yields the operative equation for time-dependent LC_{50} calculation (Equation 25, Main Manuscript):

$$LC_{50} = \frac{\sum_i M_i \cdot [i]^*}{\sum_i (M_i \cdot [i]^*)/LC_{50_i}} \tag{S19}$$
